# Critical Care: Applying Genomics to Inflammation Outcomes

**DOI:** 10.1289/ehp.113-a816

**Published:** 2005-12

**Authors:** Charles W. Schmidt

What do gunshot wounds, burns, heart attacks, arthritis, asthma, and cancer all share in common? Apart from inflicting misery, these conditions—and others too—involve inflammation, an immune response to injury and infection that normally protects, but sometimes endangers or kills patients. Caused by immune cells accumulating at a site of injury, inflammation typically guards against infection and speeds recovery; it is a critical process and, per se, does not cause disease. But unchecked inflammation that spreads or fails to subside poses chronic and acute health risks for millions of people. Asthma patients, for instance, can’t breathe because inflammatory compounds cause airway linings to swell and mucus to spread in the lungs. Inflammation also exacerbates cancer, scientists believe, by facilitating the proliferation of abnormal cells. An acute condition called sepsis—caused when infection or inflammation spills into the bloodstream—produces organ failure and shock in critically ill patients. Up to 215,000 Americans die from sepsis every year, according to the National Institute of General Medical Sciences. Worldwide, sepsis is estimated to kill 1,400 people each day, according to a consensus document published in the June 1992 issue of *Chest*.

In light of its implications, inflammation has become one of the hottest areas in biomedical research. J. Perren Cobb, a professor of surgery and genetics at Washington University in St. Louis, says a wide array of medical specialties stand to benefit from these investigations. “Inflammation is a major unifying syndrome, the investigation of which provides opportunities for multidisciplinary convergence,” he explains. “Studies of inflammation cut across all the domains at the NIH; it’s a fundamental process in human biology that ties everything together.”

Growing evidence suggests that genetic factors drive key aspects of an individual’s inflammatory outcome. Scientists studying inflammation are trying to identify the genes that drive inflammation as well as biomarkers from throughout the course of inflammation. Stephen Chanock, who heads the Section on Genomic Variation in the Pediatric Oncology Branch at the National Cancer Institute, emphasizes that the current critical care orientation of this research has broad multidisciplinary implications that extend to environmental health. “Injuries represent the ultimate gene–environment interactions,” he explains. “Usually environmental health focuses on chronic exposures, but in this case we’re studying environmental insults that are more dangerous and intense. So, the ‘environment’ in environmental health isn’t just about pollution, it’s also experiential. We’re developing practical methods for looking at inflammation that will ultimately be applied to larger public health issues.”

## Toward Better Knowledge of Inflammation

Today, genomics defines the cutting edge of inflammation research. Genomic studies, in addition to their proteomic and metabolomic cousins, aim to resolve an age-old mystery: namely, why some patients recover readily from inflammation while others suffer and die from it. The current research emphasis focuses on critical care, particularly of trauma and burn patients, who face the lethal dangers of septic complications. Ideally, new gene-based discoveries will provide diagnostic biomarkers to predict who among these patients will react poorly to inflammation and why. If doctors could reliably predict this outcome in advance, they might tailor antibiotics and other treatment options to a patient’s own inflammatory system, potentially saving lives.

Better knowledge of inflammation biology could also spawn new treatment options, Cobb says. The newest drug for sepsis—an Eli Lilly and Company product called Xigris that came on the market in 2001—helps some patients, but its cost is exorbitant: nearly $7,000 per course of treatment. What’s more, the drug reduces the risk of death by just 6% and can produce side effects such as excessive bleeding.

Among the numerous programs moving inflammation research forward is an effort funded by a National Institute of General Medical Sciences “glue grant,” so named because it “glues together” multi-disciplinary efforts to tackle biomedical questions beyond the means of any one research group. This program, called Inflammation and the Host Response to Injury, strives to determine why patients can have dramatically different outcomes after traumatic injuries and burns. Headed by Ronald Tompkins, a professor of surgery at Harvard Medical School and chief of Massachusetts General Hospital’s Burn Service, the program uses genomic and proteomic methods to study inflammation at 22 clinical centers located throughout the country. A total of $37 million was made available for the program’s first five years.

When the Inflammation and Host Response to Injury program was launched in 2001, its leaders decided to create a broad research infrastructure with uniform protocols as a first priority. “One of our first challenges was to develop guidelines, not just for the sample collection and analysis, but also for patient management,” says Lyle Moldawer, a glue grant recipient and professor of surgery at the University of Florida College of Medicine. “We recognized that all the funded centers have different protocols for the immediate care of trauma and burn patients, and we were concerned that those differences in early management might contribute to gene expression changes.”

Tompkins says creating a uniform infrastructure for the program was like building a highway. “We needed the gas stations, the on-ramps, the off-ramps,” he says. “No one had ever tried to introduce this technology into critical care medicine before.” With standard operating procedures in place and the program now in its fourth year, scientists have begun to address a subsequent challenge: extracting useful knowledge from the reams of genomic data flowing out of the program’s 22 clinics.

At the same time the glue grant program was gearing up, Cobb, senior investigator Anthony Suffredini of the NIH Critical Care Medicine Department, and Robert Danner, who heads the Infectious Diseases Section in the same department, created the Consortium for Expression Profile Studies in Sepsis specifically to identify the needs of those applying genomic methods to critical care. The consortium hosted four meetings throughout the country before evolving into the NIH Functional Genomics of Critical Illness and Injury Symposia series, which now provides a forum where glue grant recipients and others discuss research progress and results. The most recent symposium, hosted by the NIH at its Bethesda campus on 21–22 April 2005, was attended by scientists from 10 countries, all seeking to advance genomics in inflammation research.

## An Inflammation Primer

Once triggered, inflammation proceeds similarly whether caused by pollutants, pathogens, trauma, radiation, or burns. Localized mast cells in affected tissues produce histamine, a chemical mediator that dilates blood vessels at the site of injury, producing redness and heat. Histamine also renders blood vessels permeable, so leukocytes (white blood cells) can reach the injury. Leukocytes are attracted to the injury site by chemotactic proteins known as chemokines, which are secreted by endothelial cells of the blood vessels.

Leukocytes originate in bone marrow and include diverse cell types, such as neutrophils, eosinophils, basophils, monocytes, lymphocytes, and macrophages. Neutrophils arrive at the affected area first. These remarkable cells roam the body and kill pathogens on demand with a toxic blend of free radicals and protein-chewing enzymes that destroy bacterial cell walls. Monocytes engulf cellular debris and mature into macrophages, which are larger leukocytes that consume entire bacteria. These cells also secrete a variety of cytokines that recruit and activate other cell types. Lymphocytes are divided in two broad classes—B cells and T cells—each with different roles. B cells, once activated, make antibodies that attack foreign substances, while T cells kill infected cells directly.

Chemical mediators released by leukocytes during inflammation come in many varieties. Cytokines, for instance, help to regulate inflammation, whereas inter-leukins regulate T cell activity and produce systemic effects such as fever.

Normally, the whole inflammation process is self-limited and short-lived; leukocytes disperse after dispensing with infectious agents, and inflammation dies down within hours or days. Problems crop up when the response persists or spreads systemically, damaging and killing normal tissues in the process. Chronic inflammation can persist for years, causing illnesses that end with the suffix “-itis,” such as bronchitis, arthritis, and bursitis. Systemic inflammation—sepsis being one variety—occurs when cytokines reach the bloodstream and spread through the body, damaging organs far from the initial injury’s source.

## Candidate Genes

No one knows precisely what happens when inflammation goes awry. Years of immunology research have implicated hundreds of genes in abnormal inflammation, but the evidence linking them to particular outcomes is weak. Of these genes, the one coding for C-reactive protein (CRP), an acute-phase molecule whose levels shoot up during systemic inflammation, is perhaps the best known. High CRP levels are prognosticators for heart disease and stroke (which are both linked to inflammation), but its role in these conditions remains unclear. Another well-known gene—tumor necrosis factor–alpha (TNF-α)—codes for a pro-inflammatory cytokine that normally regulates leukocyte and endothelial cell activity, in addition to other functions.

By the 1990s, however, candidate gene studies had yet to produce clinical benefits for inflammation. Suffredini says scientists at the time were extremely frustrated with the lack of progress. “People were throwing up their hands and feeling [painted] into corners,” he says.

A turning point emerged at the turn of the millennium, when a rough draft of the human genome and the advent of microarrays made it possible to assess the expression of thousands of genes simultaneously. “The analogy is that for years, we’d been working on the ground to see how candidate genes interact,” Cobb explains. “But microarrays allowed us to look down at the genome from twenty thousand feet, so to speak, and that has enabled us to model much broader interactions.”

With these tools, scientists could search for entirely new genes and molecular pathways involved in disease processes. Cancer researchers were among the first to exploit the technology for clinical aims, Suffredini says, inspiring their counterparts in critical care to do the same. Thus, inflammation research entered a new phase of gene discovery that drives much of the progress in the field today. Scientists are now investigating a variation in the promoter region of TNF-α(the region that initiates protein production after binding transcription factors) that might contribute to sepsis.

Injuries represent the ultimate gene–environment interactions. Usually environmental health focuses on chronic exposures, but in this case we’re studying environmental insults that are more dangerous and intense.

–Stephen Chanock National Cancer Institute

While cancer genomics inspired similar efforts in critical care, both specialties operate under vastly different research settings. For one thing, cancer patients typically have the time and awareness to provide informed consent for blood and tissue sampling. In addition, the cohorts tend to be large and matched for age, sex, treatment history, and other parameters that can influence genomic profiles. Trauma and burn patients, on the other hand, are rushed—often unconscious—into the emergency room or intensive care unit, where live-saving treatment is the first priority. In this frenetic environment, informed consent is difficult to secure, and research sampling becomes a secondary concern.

Moreover, cancer and trauma induce totally different types of gene expression—whereas tumors typically produce localized, stable expression profiles corresponding to small portions of the genome, critical injuries trigger enormous genomic changes that affect all tissues and shift rapidly over time. Temporal factors are extremely important in critical care sampling because they have a tremendous influence on the gene profile; a sample taken 15 minutes after injury will be vastly different than one taken several hours later.

## Into the Data

According to Tompkins, investigators with the glue grant program chose to investigate normal and abnormal inflammation trajectories sequentially, each in five-year increments. Genomic and proteomic data for the normal trajectory—compiled using samples from trauma and burn patients who recovered uneventfully—are now being analyzed.

At the same time, program scientists augmented the clinical research with additional genomewide expression studies of leukocytes sampled from healthy volunteers dosed intravenously with bacterial endotoxin. These studies—which induced low-level systemic inflammation that permitted validation of sample processing protocols—enabled scientists to compare baseline and inflammatory genomic changes at varying time points. Patients weren’t harmed by the experiments, and all responses returned to normal within 24 hours.

The results, published in the 31 August 2005 issue of *Nature*, showed how complex inflammatory networks really are—between 3,000 and 5,000 genes, up to 20% of the entire genome, were activated, according to Moldawer, one of the study’s authors. “The research revealed that the magnitude of the changes was much larger than we anticipated,” he says. “We expected to see up-regulation of stress-related genes during the acute phase, but much to our surprise, the diversity of the changes was much greater than we thought it would be.”

Many of those changes, Moldawer adds, were seen in genes involved in mitochondrial energy transfer, protein synthesis, and antigen recognition—in short, biological processes that enable leukocytes to become more efficient antimicrobial agents, he says. Preliminary analyses suggested the magnitude and nature of the endotoxin response shared some similarities with the response seen in real patients. At press time, the clinical data from actual patient cohorts were still being assessed.

Although the amounts of genomic data may be computationally daunting, recent evidence from another study suggests efforts to distinguish good inflammatory outcomes from bad might have promise. This study, published in the 29 March 2005 *Proceedings of the National Academy of Sciences*, made several key discoveries. First, hospitalization and repeated sampling had only a modest effect on gene expression in healthy volunteers. Thus, the experience of being hospitalized (with its enforced bed rest and defined nutritional intake) is unlikely to influence gene expression in ways that undermine the detection of signature profiles for specific inflammatory outcomes. Second, the researchers showed that gene expression differences in whole-blood leukocytes drawn from severe trauma patients could be divided into injury-specific patterns. Taken together, says coauthor Tompkins, the findings indicate that expression profiling may yield “low-hanging fruit” in the form of highly correlated data.

## Linking Sepsis-Related Genes to Biology

Meanwhile, researchers in Germany have shown that subsets of genes can be linked directly to sepsis. Among these researchers is Trinad Chakraborty, who directs the Institute of Medical Microbiology at Justus-Liebig University. Chakraborty is completing a study of genomic factors contributing to sepsis in patients with multiple trauma or pneumonia. The study—part of a broader effort to understand why patient outcomes differ after similar injuries and illnesses—involved screening up to 20,000 genes in peripheral blood during a 14-day post-injury period. The effort, conducted in 185 patients, found 690 genes whose expression appears to correlate with sepsis. In future research, Chakraborty plans to look for single-nucleotide polymorphisms within candidate genes that predispose the sepsis phenotype, and to identify protein-based biomarkers for diagnostic use.

But Chakraborty adds that computational challenges are a serious holdup. “When we started the research, getting the microarrays to be sufficiently robust was the bottleneck,” he says. “Now we’ve resolved that problem, and bioinformatics is the bottleneck.” He and his colleagues hope to trim the 690 genes to a lesser population of 25 or so. “Then we could develop an algorithm that recognizes a profile within that smaller set of genes to indicate whether you have a likelihood of sepsis or not.”

Inflammation is a major unifying syndrome, the investigation of which provides opportunities for multidisciplinary convergence. . . .

It’s a fundamental process in human biology that ties everything together.

–J. Perren Cobb Washington University in St. Louis

U.S. scientists have also correlated genes with sepsis and used these findings to suggest a preliminary mechanism for its lethality. Led by Hector Wong, who directs the Division of Critical Care Medicine at Cincinnati Children’s Hospital Medical Center, the scientists used microarrays to compare gene profiles between children who survived sepsis and those who died from it. Children respond uniquely to sepsis in that their fatality rates are much lower than those of adults—roughly 10% compared to 30% among the latter, says Chanock.

Wong suspects that children respond better to sepsis in part because they have fewer comorbidities such as diabetes and heart disease (a status that is changing somewhat with rising childhood obesity). But he further suspects genetic factors underlie important biological differences that improve their outcomes, though at this point he can’t say how.

In recent studies presented at the April symposium, Wong found that among non-surviving children, six genes coding for metallothionein—a protein that binds zinc and removes it from the bloodstream—appeared to be highly expressed. These findings led him to a hypothesis: if severely septic children had high blood metallothionein levels, he proposed, then their blood zinc levels might be correspondingly low. “And in fact, that turned out to be true,” he says.

Another interesting finding was that the profiles showed altered expression patterns for a host of proteins that either depend on zinc or take part in zinc homeostasis. “So there’s a lot of biology there to look at,” Wong says. “We don’t know how or whether zinc is involved; there’s very little information out there about the effects of acute zinc deficiency. I find it hard to believe the foundation for sepsis is zinc, but . . . I think it can be tested.” After considering this position further, Wong adds that this is how high-throughput investigations are useful: they suggest biological mechanisms that scientists can explore further in the laboratory.

## Future Needs

Today, a genomic research culture is slowly seeping into the front lines of care for the critically ill and injured. But establishing that culture isn’t easy—emergency room and intensive care unit settings challenge researchers in many ways. Issues like informed consent for study participation and repeated intrusive blood sampling to assess temporal changes in the genome are difficult to manage, Tompkins says. Ideally, new technologies will reduce sample volume requirements, lessen the amount of time required for microarray analysis (which now averages 24 hours), and reduce microarray costs to the extent that they can be used routinely in the clinic.

We need to do a better job of educating people about the importance of this process.

–J. Perren Cobb Washington University in St. Louis

Chakraborty adds that microarray platforms need to accommodate sample degradation too. As it stands now, he says, RNA in blood samples drawn in the emergency room has a higher degradation potential than RNA in samples drawn from the more controlled environment of a research laboratory. “The platforms need to become more robust,” Chakraborty says. “That way, if the quality of the RNA drops to fifty or seventy percent rather than a hundred percent, we would still be able to get meaningful results.” Researchers with the glue grant program are also seeking to set up guidelines for standardized research procedures that will help lessen the potential for sample degradation.

Inflammation genomics also poses enormous computational challenges. Studies that lack sufficient statistical rigor are a persistent problem, Cobb says—emergency room and intensive care unit cohorts tend to be smaller than optimal, and patients come in after the trauma has occurred so they can’t serve as their own controls. At the same time, lists of inflammation-specific genes identified during microarray experiments need to be incorporated into biological models that describe their molecular interactions. Bioinformatic research and associated databases are continually advancing to meet these needs, however, and collaborations among research groups both within the United States and abroad are helping to drive the science forward.

Cobb emphasizes that despite its broad public health impact, inflammation research has yet to achieve the same public awareness as that of cancer or heart disease. “We need to do a better job of educating people about the importance of this process,” he says. This means reaching other scientists as well as the public, whose concerns often drive research funding.

In the meantime, genomic methods have generated incremental advances in our understanding of inflammation. Scientists have barely scratched the surface of its vast complexity, but perhaps in the not-too-distant future, patients will reap the benefits of their efforts.

## Figures and Tables

**Figure:**
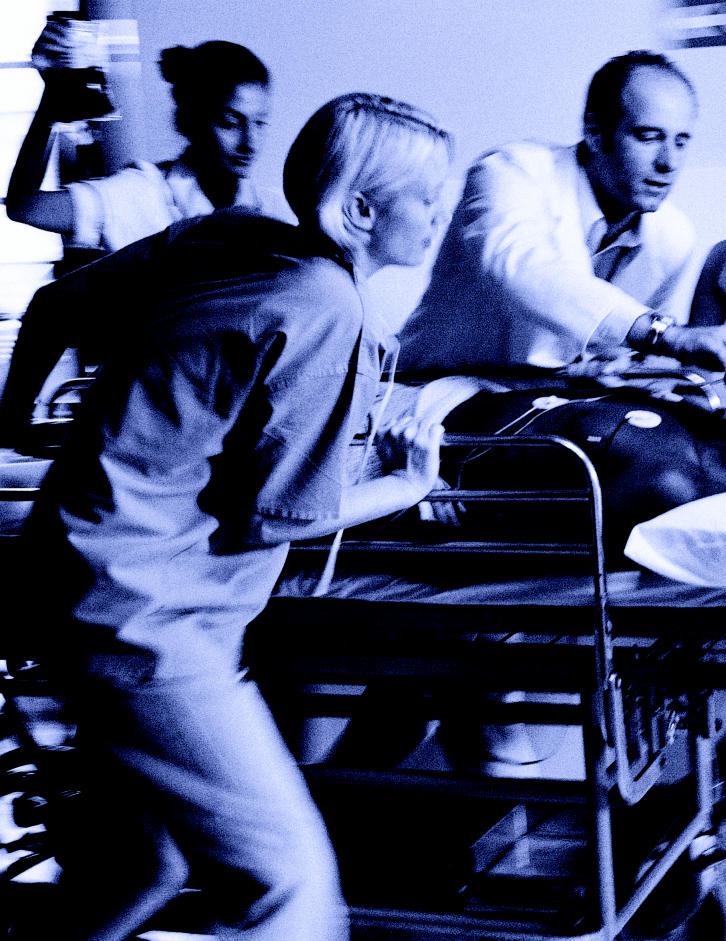


**Figure:**
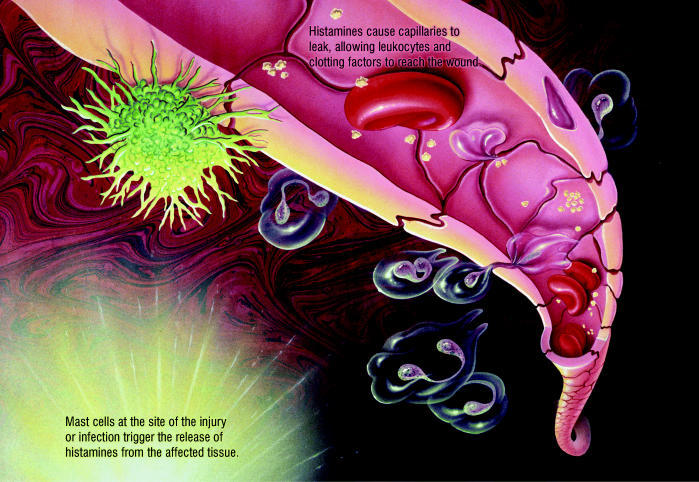
The Inflammatory Response

**Figure:**
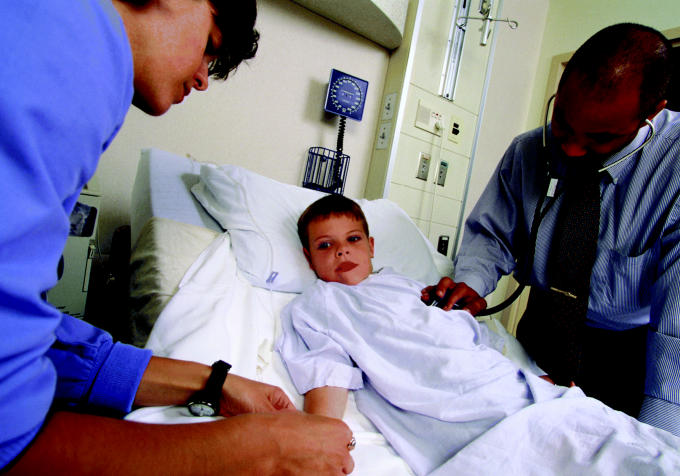
Small advances. Fatality rates from sepsis are much lower in children than adults, so much may be learned from how children’s bodies deal with inflammation.

